# The effect of systemic lidocaine on post-operative opioid consumption in ambulatory surgical patients: a meta-analysis of randomized controlled trials

**DOI:** 10.1186/s13741-021-00181-9

**Published:** 2021-04-13

**Authors:** Danielle Lovett-Carter, Mark C. Kendall, James Park, Anas Ibrahim-Hamdan, Susannah Crepet, Gildasio De Oliveira

**Affiliations:** Department of Anesthesiology, The Warren Alpert Medical School of Brown University, 593 Eddy Street, Providence, RI 02903 USA

**Keywords:** Lidocaine, Postoperative opioid consumption, Acute pain, Meta-analysis

## Abstract

**Background:**

Ambulatory surgical procedures continue to grow in relevance to perioperative medicine. Clinical studies have examined the use of systemic lidocaine as a component of multimodal analgesia in various surgeries with mixed results. A quantitative review of the opioid-sparing effects of systemic lidocaine in ambulatory surgery has not been investigated. The primary objective of this study was to systematically review the effectiveness of systemic lidocaine on postoperative analgesic outcomes in patients undergoing ambulatory surgery.

**Methods:**

We performed a quantitative systematic review of randomized controlled trials in electronic databases (Cochrane Library, Embase, PubMed, and Google Scholar) from their inception through February 2019. Included trials investigated the effects of intraoperative systemic lidocaine on postoperative analgesic outcomes, time to hospital discharge, and adverse events. Methodological quality was evaluated using Cochrane Collaboration’s tool and the level of evidence was assessed using GRADE criteria. Data was combined in a meta-analysis using random-effects models.

**Results:**

Five trials evaluating 297 patients were included in the analysis. The pooled effect of systemic lidocaine on postoperative opioid consumption at post-anesthesia care unit revealed a significant effect, weighted mean difference (95% CI) of − 4.23 (− 7.3 to 1.2, *P* = 0.007), and, at 24 h, weighted mean difference (95% CI) of − 1.91 (− 3.80 to − 0.03, *P* = 0.04) mg intravenous morphine equivalents. Postoperative pain control during both time intervals, postoperative nausea and vomiting reported at post anesthesia care unit, and time to hospital discharge were not different between groups. The incidence rate of self-limiting adverse events of the included studies is 0.007 (2/297).

**Conclusion:**

Our results suggest that intraoperative systemic lidocaine as treatment for postoperative pain has a moderate opioid-sparing effect in post anesthesia care unit with limited effect at 24 h after ambulatory surgery. Moreover, the opioid-sparing effect did not impact the analgesia or the presence of nausea and vomiting immediately or 24 h after surgery. Clinical trials with larger sample sizes are necessary to further confirm the short-term analgesic benefit of systemic lidocaine following ambulatory surgery.

**Trial registration:**

PROSPERO (CRD42019142229)

**Supplementary Information:**

The online version contains supplementary material available at 10.1186/s13741-021-00181-9.

## Background

Postoperative pain, along with nausea, remains one of the most common reasons cited for delay in discharge and unplanned admission after ambulatory surgery (Shirakami et al., 2005; McGrath et al., 2004; Rawal, 2007). Outpatient surgery presents a challenge, as analgesic options matching the potency of opioids are limited. Though powerful analgesics, opioids are also known for their adverse effects such as nausea, vomiting, sedation, and hypoventilation, which may delay recovery or result in unplanned admissions. A recent study demonstrated that higher opioid administration peri-operatively was associated with an increase in re-admission rates (Long et al., 2018). The use of multimodal analgesia to reduce opioid requirements for pain management has been proposed to offset this issue with some success (Duncan et al., 2019; Stundner et al., 2019).

Lidocaine is a local anesthetic that, when given systemically at a rate of 1.5–3 mg kg^−1^ h^−1^, has been shown analgesic and anti-inflammatory properties (McCarthy et al., 2010; Hermanns et al., 2019). Several recent studies have demonstrated intravenous lidocaine as an effective adjunct in the management of post-operative pain of surgical inpatients (Vigneault et al., 2011; Sakata et al., 2020).The benefits of systemic lidocaine include reduction in postoperative pain, decrease opioid requirements, and a decrease in hospital length of stay. Recent literature revealed favorable results for the use of systemic lidocaine in patients undergoing laparoscopic cholecystectomy and open abdominal surgery (Zhao et al., 2018; Marret et al., 2008). A quantitative review investigating the opioid-sparing effects of systemic lidocaine on postoperative pain in ambulatory surgery has not been performed.

The primary aim of this study was to systematically review the effect of systemic lidocaine on postoperative opioid consumption in ambulatory surgical patients. Secondary outcomes assessed were pain scores in the post anesthesia care unit (PACU), adverse events related to treatment, and time to discharge readiness. We hypothesize that, in line with previous studies of inpatients, intraoperative administration of intravenous lidocaine will be effective in reducing opioid consumption in the postoperative period in patients undergoing ambulatory surgery.

## Methods

The review was performed in compliance with the PRISMA statement (Moher et al., 2009). The systematic review was registered in the international database PROSPERO (CRD42019142229). Institutional review board approval and oversight was not required. We followed similar methods as previously published by our investigators (Kendall et al., 2020; Lovett-Carter et al., 2019).

### Systematic search strategy

Peer-reviewed studies exploring the effectiveness of systemic lidocaine to control (normal saline infusion) on postoperative surgical analgesia following ambulatory surgery were searched using electronic databases (PubMed, Embase, Cochrane, and Google Scholar) from inception up to February 2019. Using free text, the search words “systemic lidocaine,” “intravenous lidocaine,” “ambulatory,” and “outpatient” were used in various combinations and presented in Additional file 1. The search was restricted to adults 18 years of age or older, and no language restrictions were applied. In addition, the bibliographies from the identified articles, reviews, and meta-analyses were also reviewed for additional studies. Unpublished and non-peer reviewed studies were not investigated.

### Selection criteria

The inclusion/exclusion criteria were defined prior to the implementation of the systematic review. We included randomized control trials that compared intravenous lidocaine given intraoperatively with or without bolus to control in patients undergoing ambulatory surgery. The duration of lidocaine infusion had to continue at least until the end of surgery. The control group was defined as patients who received intraoperative normal saline via infusion. Articles had to describe postoperative outcomes of either pain scores or opioid consumption. Studies were excluded from analysis if patients were undergoing planned admission postoperatively or a direct comparison between systemic lidocaine and control group could not be determined. Non-randomized controlled trials, case reports, or editorials were not considered for inclusion. No minimum sample size was required for inclusion in the quantitative analysis.

### Selection of included articles and data extraction

Two investigators (DLC and MCK) independently reviewed the abstracts and trial outcomes of the 372 articles retrieved by the initial query. Articles that did not fulfill the inclusion criteria or met the exclusion criteria were omitted. Disputes between the reviewers were finalized by discussion, and if a resolution was not met, the final decision was determined by an additional investigator (GDO).

The data from each individual trial was extracted and recorded on a collection form. The variables were extracted from the text or tables, and where data was not available, it was obtained directly from the figures. The variables extracted from the trials included sample size, number of patients in the intervention and control groups, type of surgery, systemic lidocaine/control infusion rate/dose, time to meet hospital discharge readiness (min), postoperative opioid consumption, postoperative pain scores at rest, and adverse events associated with the intervention. The numerical rating scale of pain or visual analog scale were adapted to an 11-point numeric rating scale (0 = no pain, 10 = extreme pain). Postoperative opioids were converted to intravenous morphine equivalents.

Continuous outcomes were recorded using mean and standard deviation. Variables presented as median, interquartile range, or mean ± 95% confidence interval (CI) were converted to mean and standard deviation (Hozo et al., 2005; Wan et al., 2014). For studies that did not provide standard deviation, the standard deviation was estimated using the most extreme values. If the same outcome variable was reported more than once, then the most conservative value was used.

### Outcomes

The primary outcome was postoperative opioid consumption (IV morEq) reported at 24 h following surgery. Secondary outcomes included postoperative pain scores (numeric pain rating score, 0 = no pain, 10 = extreme pain) at PACU and at 24 h after surgery, incidence of postoperative nausea and vomiting (*n*), time to discharge (min), and adverse events (i.e., arrhythmias).

### Bias assessment

The Cochrane Risk-of-Bias Tool was used to evaluate the potential risk of bias in the included randomized trials. The risk bias tool involves six specific domains involving selection bias, detection bias, performance bias, attrition bias, reporting bias, and other potential source of bias (Higgins et al., 2011). Two investigators (DCL and MCK) individually evaluated the risk of bias of the included studies. An additional investigator was involved in the assessment if there was a discrepancy among the previous two investigators (GDO). The assessment of each domain was recorded either as low risk, high risk, or unclear risk.

### Meta-analysis

The pooled data consisting of continuous variables (total opioid consumption at 24 h, pain score (NRS) at PACU and at 24 h) was calculated and expressed as weighted mean differences (WMD) with 95% confidence intervals. Dichotomous outcomes (i.e., side effects) were reported as odds ratio with 95% confidence intervals. Differences were considered statistically significant when the *P* value was < 0.05 and the 95% CI for continuous data did not include zero or one for dichotomous data. Owing to the limited amount of randomized trials identified, we elected to use the random-effects model in an attempt to generalize our findings to trials not included in our meta-analysis (DerSimonian & Laird, 2015).

The risk of publication bias was investigated by examining for asymmetric funnel plots using Egger’s regression test (Egger et al., 1997). In the presence of an asymmetric funnel plot, a file drawer analysis was performed. This test estimates the lowest number of additional studies that if they would become available, it would reduce the combined effect to non-significance assuming the average *z* value of the combined *P* values of these missing studies would be 0 (Bradley & Gupta, 1997).

Heterogeneity was considered moderate if the *I*^2^ statistic was in the range of 30 to 60%. If heterogeneity was high in the included studies, we performed a sensitivity analysis by removing individual studies and examining its effect on the overall heterogeneity. A *P* value < 0.05 was required to reject the null hypothesis. All statistical analysis was performed by Comprehensive Meta-analysis software version 3 (Biostat, Englewood, NJ) and Stata 15 (StataCorp LP, College Station, Texas).

## Results

**Fig. 1:**
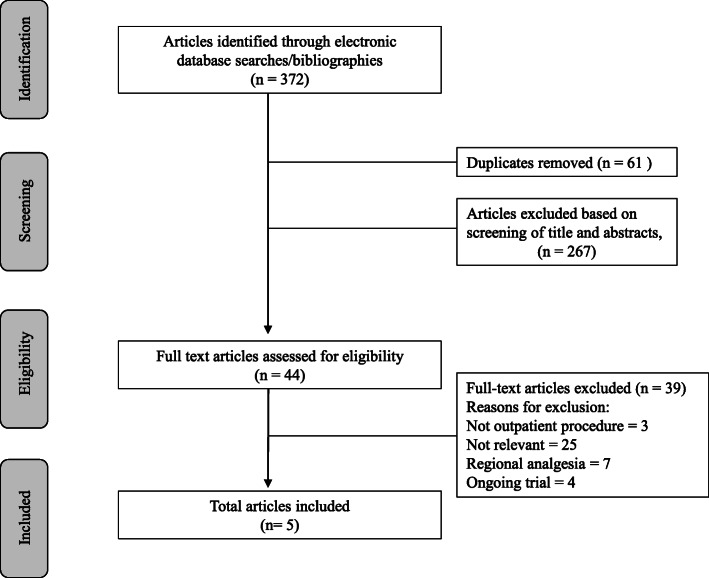
Flow chart outlining systematic review of randomized controlled trials

The primary search yielded 372 studies and, after screening and removing article duplications, 44 potential articles were identified. Articles that did not meet eligibility upon further review of full texts were excluded. The specific reasons for exclusions of articles that were fully reviewed are shown in Fig. 1. A total of 5 randomized trials with 297 patients met the inclusion criteria, and the characteristics of the studies are summarized in Table 1. The median and interquartile range of the sample size for included studies was 58 (49 to 70). All 5 randomized controlled trials described postoperative opioid consumption and/or pain scores at rest (De Oliveira et al., 2012; Dewinter et al., 2016; Lauwick et al., 2008; McKay et al., 2009; Ortiz et al., 2016).

**Table 1:** Summary of included randomized controlled trials

Author	Year	Procedure	Number treatment/control	Intervention/control	Infusion duration	Type of anesthesia	Adverse events
De Oliveira et al. (De Oliveira et al., 2012)	2012	Gynecological laparoscopy	35/35	1.5 mg/kg lidocaine bolus with 2 mg/kg/h infusion 0.9% normal saline	End of surgery	Sevoflurane	None
Dewinter et al. (Dewinter et al., 2016)	2016	Laparoscopic sterilization	39/40	1.5 mg/kg lidocaine bolus with 1.5 mg/kg/h infusion 0.9% normal saline	30 min after surgery	Sevoflurane	None
Lauwick et al. (Lauwick et al., 2008)	2008	Laparoscopic cholecystectomy	25/24	1.5 mg/kg lidocaine bolus with 1.5 mg/kg/h infusion 0.9% normal saline	End of surgery	Desflurane	(1) Persistent hypertension, (1) rapid atrial fibrillation
McKay et al. (McKay et al., 2009)	2009	Laparoscopic endocrine/breast/gynecology/minor orthopedic	29/27	1.5 mg/kg lidocaine bolus with 2 mg/kg/h infusion 1.5 mg/kg lidocaine bolus 0.9% normal saline infusion	60 min after surgery	Sevoflurane Desflurane Isoflurane	(1) Dizziness and visual disturbances
Ortiz et al. (Ortiz et al., 2016)	2016	Laparoscopic cholecystectomy	21/22	1.5 mg/kg lidocaine bolus with 3 mg/kg/h infusion 0.9% normal saline infusion	60 min after surgery	Isoflurane	None

### Quality assessment

All trials reported inclusion and exclusion criteria and described baseline characteristics. Randomized treatment allocation sequences were created with number generator computer software programs or random number tables in all studies. Randomized controlled trials describing proper concealment of treatment allocation were described in 4 trials (De Oliveira et al., 2012; Dewinter et al., 2016; Lauwick et al., 2008; McKay et al., 2009). All but one study described study personnel and outcome assessors as blinded to treatment allocation (Ortiz et al., 2016).The description of patient blinding was clear in all studies. The methodological quality and judgments about each risk of bias domain as a percentage across all included studies are presented in Table 2. The quality of evidence of the included studies was summarized using the Grading of Recommendations, Assessment, Development, and Evaluation (GRADE) criteria and is presented in Table 3 (Guyatt et al., 2008).

**Table 2:**
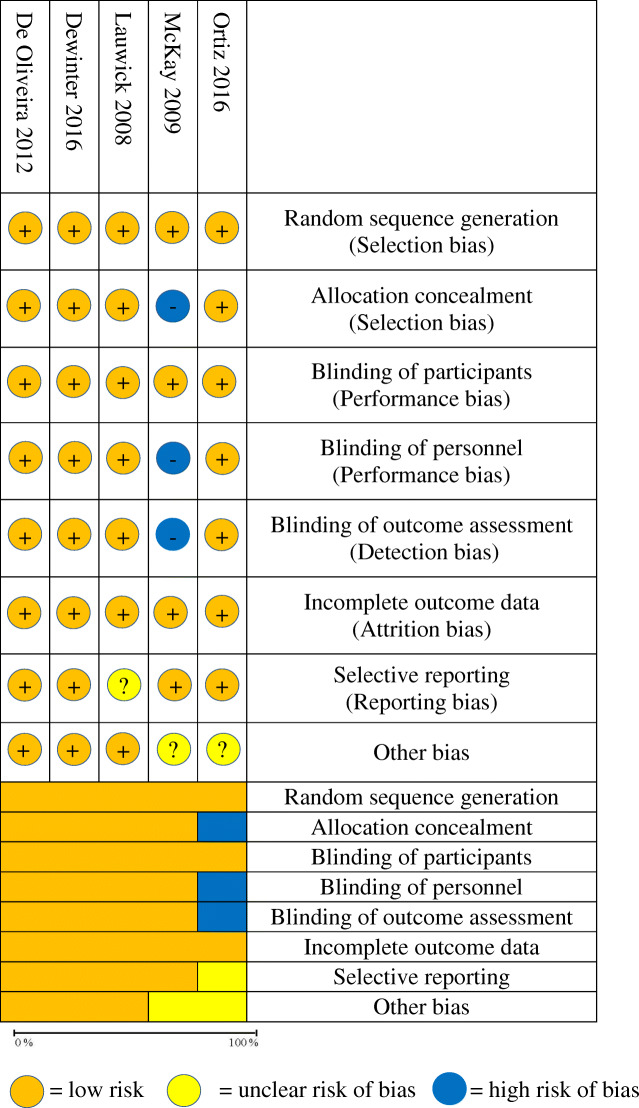
Methodological quality and risk bias summary

**Table 3:** Summary of the quality of evidence (GRADE) for comparing systemic lidocaine to a control group for the primary and secondary outcomes of the included studies

# Studies in design (n)	Risk of bias	Inconsistency	Indirectness	Imprecision	Publication bias	Overall quality of evidence^d^
Postoperative opioid consumption at 24 h						
5 (297)	Not serious^a^	Not serious	Not serious	Serious^b^	Undetected	⨁⨁⨁◯ Moderate
Postoperative opioid consumption at PACU						
3 (169)	Not serious^a^	Not serious	Not serious	Serious^b^	Detected^c^	⨁⨁◯◯ Low
Postoperative pain at rest at 24 h						
4 (218)	Not serious^a^	Not serious	Not serious	Serious^b^	Undetected	⨁⨁⨁◯ Moderate
Postoperative pain at rest at PACU						
4 (218)	Not serious^a^	Not serious	Not serious	Serious^b^	Undetected	⨁⨁⨁◯ Moderate
Postoperative nausea and vomiting						
4 (254)	Not serious^a^	Not serious	Not serious	Serious^b^	Undetected	⨁⨁⨁◯ Moderate

^a^Majority of studies had allocation concealment and used blinded outcome assessments; lost to follow-up was very low; the overall risk of bias was felt to be not serious

^b^Imprecise due to wide confidence interval; few numbers of events

^c^Egger’s regression test revealed a one-sided *P* = 0.03

^d^Grade Workshop Group grades of evidence: high quality, further research very unlikely to change confidence in estimate of effect; moderate quality, further research likely to have important impact on confidence in estimate of effect and may change estimate; low quality, further research very likely to have important impact on confidence in estimate of effect and likely to change estimate; very low quality, very uncertain about estimate

### Postoperative opioid consumption at 24 h after surgery

**Fig. 2:**
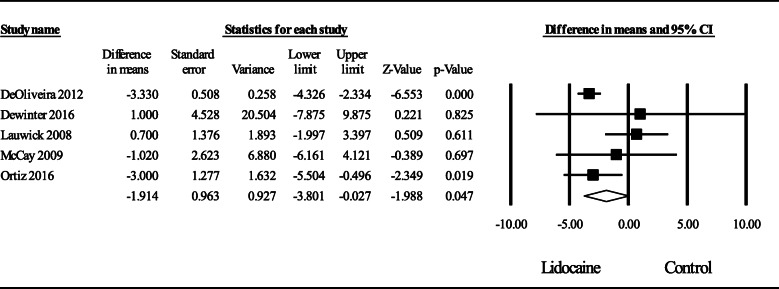
Meta-analysis evaluating the effect of intraoperative lidocaine infusion on postoperative opioid consumption compared to control at 24 h after ambulatory surgery. The overall effect of intravenous lidocaine versus control was estimated as a random effect. The point estimate (95% confidence interval) for the overall effect was − 1.91 (− 3.80 to − 0.03) (*P* = 0.04) mg intravenous morphine equivalents

Moderate quality evidence from the pooled data of 5 RCTs (De Oliveira et al., 2012; Dewinter et al., 2016; Lauwick et al., 2008; McKay et al., 2009; Ortiz et al., 2016) investigating the effect of intravenous lidocaine on postoperative opioid consumption compared to control at 24 h revealed a significant effect with a weighted mean average WMD (95% CI) of − 1.91 (− 3.80 to − 0.03) mg intravenous morphine equivalents (*P* = 0.04) (Fig. 2). Heterogeneity was moderate, *I*^2^ = 54%. A sensitivity analysis by removing individual studies did not substantially reduce heterogeneity. An analysis of the funnel plot did not reveal asymmetry; Egger’s regression test revealed a one-sided *P* = 0.10.

### Postoperative opioid consumption at PACU

**Fig. 3:**
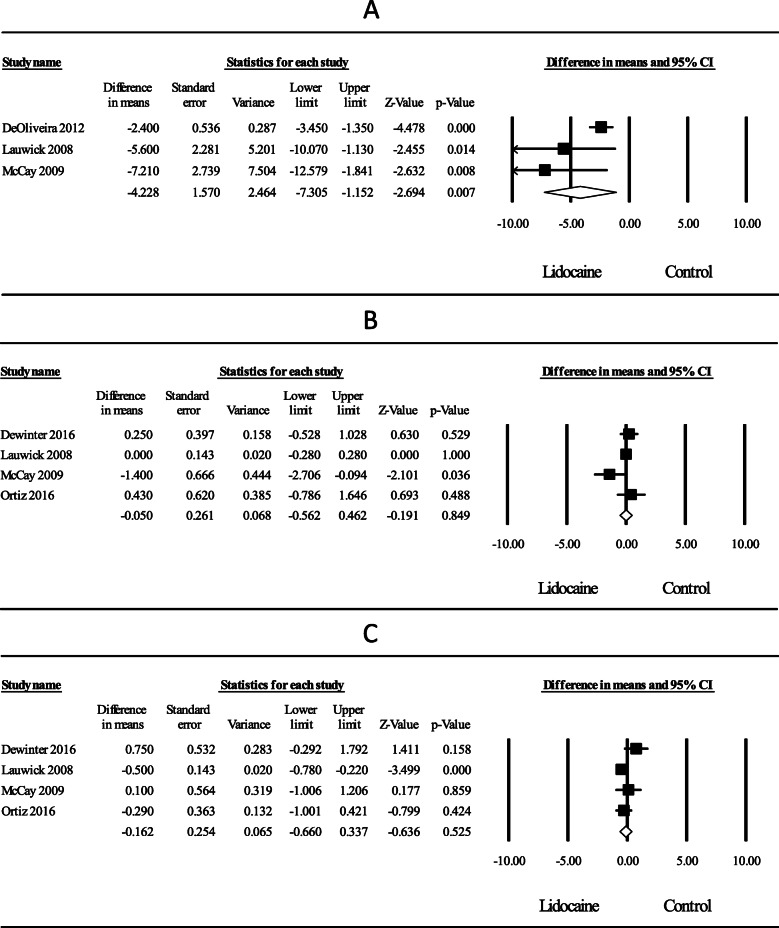
Meta-analysis evaluating the effect of intraoperative intravenous lidocaine on **a** postoperative opioid consumption at PACU and postoperative pain at rest at **b** PACU and **c** 24 h after surgery. In part **a**, the point estimate (95% confidence interval) for the overall effect was − 4.23 (− 7.30 to − 1.15) (*P* = 0.007) mg intravenous morphine equivalents. In part **b**, the point estimate (95% confidence interval) for the overall effect on postoperative pain at PACU was − 0.05 (− 0.56 to 0.46) (*P* = 0.85) whereas in part **c**, the point estimate for the overall effect on postoperative pain at 24 h was − 0.16 (− 0.66 to 0.34) (*P* = 0.53) (0–10 numerical scale). The weighted mean difference for individual studies is represented by the square symbol on forest plot, with 95% CI of the differences shown as a solid line. The overall effect was estimated as a random effect

Low-quality evidence from three RCTs (De Oliveira et al., 2012; Lauwick et al., 2008; McKay et al., 2009) investigating the effect of intravenous lidocaine on postoperative opioid consumption at PACU compared to control revealed a significant effect with a weighted mean average WMD (95% CI) of − 4.23 (− 7.30 to − 1.15) mg intravenous morphine equivalents (*P* = 0.007) (Fig. 3a). Heterogeneity was also moderate, *I*^2^ = 57%.

### Postoperative pain at PACU after surgery

Moderate quality evidence from the pooled data of four RCTs (De Oliveira et al., 2012; Lauwick et al., 2008; McKay et al., 2009; Ortiz et al., 2016) examining intravenous lidocaine on postoperative pain compared to control at PACU failed to reveal a significant difference, WMD (95% CI) of − 0.05 (− 0.56 to 0.46); *P* = 0.84 (Fig. 3b). Statistical heterogeneity was moderate, *I*^2^ = 43%. An examination of the funnel plot did not reveal asymmetry; Egger’s regression test revealed a one-sided *P* = 0.37. A sensitivity analysis by deleting individual studies did not substantially reduce heterogeneity.

### Postoperative pain at 24 h after surgery

Moderate quality evidence of four RCTs (De Oliveira et al., 2012; Lauwick et al., 2008; McKay et al., 2009; Ortiz et al., 2016) evaluating the effect of intravenous lidocaine on postoperative pain compared to control at 24 h failed to reveal a statistically significant effect, WMD (95% CI) of − 0.16 (− 0.66 to 0.34); *P* = 0.53 (Fig. 3c). Statistical heterogeneity was moderate, *I*^2^ = 50%. An exploration of the funnel plot did not reveal asymmetry; Egger’s regression test revealed a one-sided *P* = 0.06. A sensitivity analysis by deleting individual studies did not noticeably reduce heterogeneity.

### Time to meet hospital discharge readiness

**Fig. 4:**
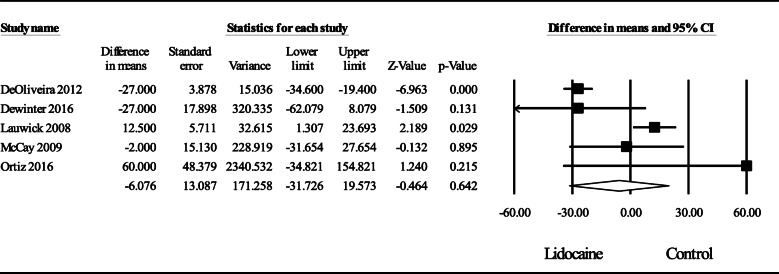
Meta-analysis evaluating the effect of intraoperative intravenous lidocaine on time to hospital discharge readiness compared to control after ambulatory surgery. The overall effect of systemic lidocaine versus control was estimated as a random effect. The point estimate (95% confidence interval) for the overall effect was − 6.08 (− 31.73 to 19.57) (*P* = 0.64) minutes

The pooled data of all 5 studies (De Oliveira et al., 2012; Dewinter et al., 2016; Lauwick et al., 2008; McKay et al., 2009; Ortiz et al., 2016) evaluating the result of intravenous lidocaine on time to hospital discharge readiness compared to control after surgery did not reveal a statistically significant effect, WMD (95% CI) of − 6.08 (− 31.73 to 19.57); *P* = 0.64 (Fig. 4). Statistical heterogeneity was substantial, *I*^2^ = 88%. An exploration of the funnel plot did not reveal asymmetry; Egger’s regression test revealed a one-sided *P* = 0.28. A sensitivity analysis by deleting individual studies did not substantially reduce heterogeneity.

### Postoperative side effects

**Fig. 5:**
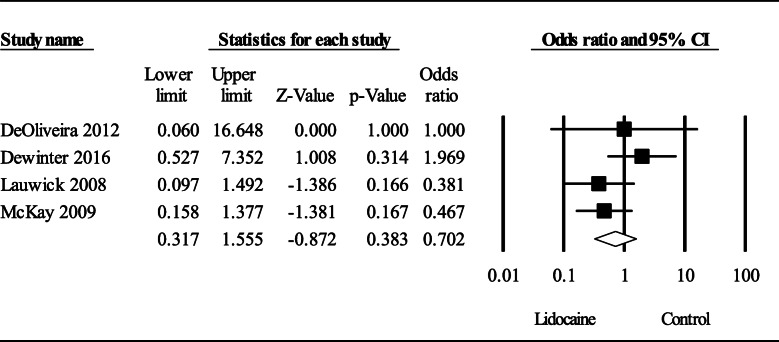
Meta-analysis evaluating the effect of systemic lidocaine on the occurrence of nausea and vomiting compared to control at PACU after ambulatory surgery. Squares to the left of the middle vertical line indicates that intraoperative intravenous lidocaine is associated with decreased odds of nausea and vomiting although not statically significant (*P* = 0.70). The horizontal lines represent the 95% CI and the diamond shape represents the overall effect of systemic lidocaine compared to control. CI = confidence interval

Moderate quality evidence of 4 RCTs (De Oliveira et al., 2012; Dewinter et al., 2016; Lauwick et al., 2008; McKay et al., 2009) did not suggest that intravenous lidocaine had a significant effect on postoperative nausea and vomiting compared to control at PACU, OR (95% CI) of 0.70 (0.32 to 1.56); *P* = 0.38 (Fig. 5). Heterogeneity was low (*I*^2^ = 19%).

### Adverse events

Two studies reported self-limiting adverse events following intraoperative lidocaine infusion. In one study, a patient experienced persistent hypertension and atrial fibrillation following the administration of systemic lidocaine (Lauwick et al., 2008). In the second study, a patient reported lightheadedness coupled with dizziness that resolved after discontinuing the lidocaine infusion (McKay et al., 2009). None of the patients allocated to the control group of the selected studies reported any self-limiting adverse events. The incidence rate of self-limiting adverse events of the included studies is 0.007 (2/297).

## Discussion

Our results suggest that intraoperative lidocaine infusion as treatment for post-operative pain has a moderate opioid-sparing effect in the post-anesthesia care unit with a limited effect at 24 h after ambulatory surgery. The pooled results of these trials support its use as part of a multimodal regimen in outpatient surgery. However, the use of lidocaine infusion for postoperative pain control beyond the immediate postoperative period remains uncertain. This finding is clinically significant as it is, to the best of our knowledge, the first meta-analysis that has demonstrated the opioid-sparing effects of lidocaine in an exclusively ambulatory population.

The reason why lidocaine reduces postoperative opioid consumption is likely multifactorial. The proposed mechanisms of analgesia include a reduction in tonic neural discharge of active peripheral nerve fibers (Chabal et al., 1989; Woolf & Wiesenfeld-Hallin, 1985) as well as a selective depression of pain transmission in the spinal cord (Juhlin, 1986; Tanelian & MacIver, 1991). Our findings of opioid reduction in this population are in line with recent a meta-analysis of eight RCTs investigating systemic lidocaine use in laparoscopic cholecystectomy (Zhao et al., 2018). Furthermore, recent literature demonstrated lower pain scores, less PONV, reduced duration of ileus, and a shorter hospital stay in patients receiving lidocaine, undergoing major abdominal surgery (Marret et al., 2008). Farag and colleagues reported that in 116 patients undergoing complex spine surgery, patients that received lidocaine infusion from induction to 8 h after surgery had a 25% reduction in opioid consumption at 48 h (Farag et al., 2013).

Postoperative nausea and vomiting are known side effects related to systemic use of morphine. In our study, systemic lidocaine did not decrease the occurrence of nausea and vomiting compared to control at PACU. We also did not find a reduction of pain scores at rest either at PACU or at 24 h after surgery. This contrasts with the findings of other studies where they demonstrated an overall reduction in VAS scores post-operatively (Zhao et al., 2018; Marret et al., 2008). It is possible that the duration of the infusion may have played a role in these findings. Many studies have measured lidocaine plasma concentrations and used this data to quantify therapeutic and toxic levels; however, the results have been conflicting. Martin et al., who found no benefit of lidocaine infusion in hip fracture surgeries, also recognized the short infusion time as a potential limitation of the benefits of IV lidocaine (Martin et al., 2008). However, Koppert and colleagues showed positive results with a similar length low dose lidocaine infusion where plasma concentrations were similar to or lower than Martin et al. (Koppert et al., 2004). Some authors have proposed caution regarding optimal timing and dose, which many believe has not been clearly established (McCarthy et al., 2010; Weibel et al., 2018). Given conflicting reports regarding optimal length of infusion and total dose in multiple previous studies, it remains unclear what effect, if any, these factors have on outcomes. Moreover, the analgesic and anti-emetic adjuncts may have rendered any minor benefits undetectable within a small sample size.

We found no significant difference between groups in time to discharge readiness, which was not unexpected. It has been recognized in previous studies that intravenous lidocaine appears to benefit patients undergoing bowel surgery to a greater degree. The benefits of lidocaine in abdominal surgery are likely related, not only to the factors affecting nociceptive transmission as discussed above, but also to the multifactorial nature of gastrointestinal dysfunction and ileus (McCarthy et al., 2010). Lidocaine has been shown to reduce ileus (Zhao et al., 2018; Marret et al., 2008), which prolongs hospital length of stay and contributes to nausea and patient discomfort. In this regard, lidocaine may play a more significant role in patients undergoing laparoscopic ambulatory surgery. The variety of surgeries included in our cohort as well as the relatively small sample size may have obscured any potential benefit in reducing hospital length of stay. Additionally, these proposed benefits may be insignificant in this context given the fact that such surgeries involve minimal disruption of bowel.

Our results demonstrate that the effect of intravenous lidocaine on adverse events compared to control is promising although uncertain. Our findings are similar to those reported by Zhao and colleagues which reported no occurrence of systemic toxicity in patients receiving intraoperative lidocaine infusion. One trial investigating intraoperative lidocaine infusion in patients undergoing abdominal surgery reported one patient who experienced a cardiac arrhythmia with otherwise stable vital signs and no further complications (Marret et al., 2008).

Local anesthetic systemic toxicity (LAST) remains a major concern for many practitioners and, while it has a high morbidity, fortunately, it remains a rare entity (Neal et al., 2018). Lidocaine infusions have been safely used since the 1960s for chronic pain and diabetic nephropathies (Edwards, 1999; Bach et al., 1990). While there are case reports of lidocaine infusions causing toxicity, infusions are significantly less likely to cause LAST compared to other procedures (e.g., peripheral nerve blocks and neuraxial anesthesia techniques) that are performed far more commonly in anesthesia departments (Gitman & Barrington, 2018). Some strategies to prevent LAST have been proposed including avoidance or caution with dose in patients at extremes of age and those with lower muscle mass, significant cardiac ischemia, conduction abnormalities, or liver disease (Neal et al., 2018).

The findings of our study should only be interpreted within the context of its limitations. First, we limited our comparsion to acute postoperative pain. Several authors have investigated the use of intravenous lidocaine at higher doses for the treatment of neuropathic pain and have observed that such use confers additional benefits in these patients (Tremont-Lukats et al., 2005; Bailey et al., 2018; Kranke et al., 2015). However, the relatively low rate and short duration of lidocaine infusions used in our study population, while similar across studies included in this analysis, are not expected to have an effect on pre-exisiting periphral neuropathic pain. Second, we included a variety of different ambulatory surgical procedures in an attempt to improve the generalizablility of our findings, which likely contributed to the heterogenity observed in the results of the current studies. Nonetheless, we used the random effect model for all of the analyses. Third, not all ambulatory surgical patients may be candidates for intravenous lidocaine. Last, we were not able to examine the outcomes of patient satisfaction or pain with activity as it was not recorded in the available studies.

## Conclusion

In summary, the use of systemic lidocaine revealed a moderate opioid sparing effect in PACU and sparse clinical effect at 24 h after ambulatory surgery. In addition, the opioid sparing effect of lidocaine did not demonstrate an effect on pain scores or the presence of nausea and vomiting at PACU or 24 h after surgery. The use of systemic lidocaine as a non-opioid analgesic should be considered as part of a multimodal regimen to decrease postoperative opioid consumption in the acute postoperative period. Further investigations with large sample sizes evaluating the effect of systemic lidocaine on analgesic outcomes on ambulatory surgical patients are warranted with emphasis on duration of the infusion and its effects on postoperative quality of life.

## Supplementary Information

**Additional file 1: Appendix A.** Search strategy

## Data Availability

The datasets during and/or analyzed during the current study available from the corresponding author on reasonable request.

## Abbreviations

CI: Confidence interval
GRADE: Grading of Recommendations, Assessment, Development, and Evaluation
*I*^2^: Heterogeneity
IQR: Interquartile range
IV: Intravenous
LAST: Local anesthetic systemic toxicity
NRS: Numerical rating scale
PACU: Post anesthesia care unit
PONV: Postoperative nausea and vomiting
PRISMA: Preferred Reporting Items for Systematic Reviews and Meta-Analyses
RCT: Randomized controlled trial
VAS: Visual analog scale pain
WMD: Weighted mean differences
