# Supplementary material for: The effect of systemic lidocaine on post-operative opioid consumption in ambulatory surgical patients: a meta-analysis of randomized controlled trials
Source: Perioper Med (Lond). 2021 Apr 13;10:11. doi: 10.1186/s13741-021-00181-9 (PMC8042682; doi:10.1186/s13741-021-00181-9)
Supplement: Supplementary file 1 — Additional file 1: Appendix A. Search strategy [file 13741_2021_181_MOESM1_ESM.docx]

**Appendix A**: Search strategy

*PubMed*

Search ((((((systemic lidocaine) OR intravenous lidocaine) AND Randomized Controlled Trial[ptyp] AND Humans[Mesh] AND adult[MeSH])) AND ( ( Randomized Controlled Trial[ptyp] OR Clinical Trial[ptyp] ) AND Humans[Mesh] AND adult[MeSH]))) AND ambulatory Sort by: Best Match Filters: Randomized Controlled Trial; Clinical Trial; Humans; Adult: 19+ years

*Embase*

(('intravenous lidocaine' OR (intravenous AND ('lidocaine'/exp OR lidocaine)) OR 'systemic lidocaine':jt) AND outpatient:ab,ti OR ambulatory:af) AND [randomized controlled trial]/lim

*Cochrane*

#1 MeSH descriptor: [Lidocaine] explode all trees

#2 (ambulatory):ti,ab,kw

#3 (outpatient):ti;ab,kw

#4 #2 or #3

#5 #1 and #4

#8 systemic

#9 intravenous

#10 #6 or #7

#11 #5 and #8

Google Scholar limited to first 125 results
